# Immunotoxin – a new treatment option in patients with relapsed and refractory Hodgkin lymphoma

**DOI:** 10.1515/raon-2015-0036

**Published:** 2015-11-27

**Authors:** Barbara Jezersek Novakovic

**Affiliations:** Division of Medical Oncology, Institute of Oncology Ljubljana, Ljubljana, Slovenia

**Keywords:** Hodgkin lymphoma, relapsed and refractory, new treatment option, treatment effectiveness, toxicity

## Abstract

**Background:**

Even though Hodgkin lymphoma is a highly curable disease, some of the patients have either a refractory disease or experience a relapse following a successful primary therapy. Durable responses and remissions in patients with relapsed or refractory disease may be achieved in approximately one-half with salvage chemotherapy followed by high dose chemotherapy (HDT) and autologous hematopoietic cell rescue (SCT). On the other hand, patients who relapse after HDT and autologous SCT or those who have failed at least two prior multi-agent chemotherapy regimens and are not candidates for HDT have limited treatment options.

**Conclusions:**

A new treatment option in this population is an immunotoxin Brentuximab vedotin composed of a CD30 directed antibody linked to the antitubulin agent monomethyl auristatin E. It has demonstrated a substantial effectiveness and an acceptable toxicity. In the pivotal study, the overall response rate was 75% with 34% of complete remissions. The median durations of response were 20.5 and 6.7 months for those with complete remission and all responding patients, respectively. The median overall survival was 40.5 months (3-years overall survival 54%) and the median progression-free survival 9.3 months. The most common non-hematologic toxicities were peripheral sensory neuropathy, nausea, and fatigue while the most common severe side effects were neutropenia, thrombocytopenia, anemia, and peripheral sensory neuropathy.

## Introduction

Hodgkin lymphoma (HL) is a highly curable disease. However, some of the patients have either a primary refractory disease or experience a relapse following a successful initial therapy.^1^–^5^ Primary refractory disease refers to those patients who do not achieve a complete remission after initial therapy. The incidence of primary refractory disease varies depending upon the stage of disease at diagnosis and the treatment regimen used and it occurs in approximately 10 to 15% of patients undergoing primary treatment.^4^–^6^ The likelihood of relapse of HL from initial therapy in the era of systemic or combined modality therapy is approximately 10 to 15% for localized HL and 20 to 40% for advanced disease, dependent on prognostic factors. Approximately one-half of these relapses occur in the first 12 months from induction and an additional one-quarter occurs at one to three years thereafter. Late relapses (*i.e.,* more than 3 years following treatment) occur at the rate of a few percent per year, extending up to 12 years post-treatment.^1^–^3^

Durable responses and remissions in patients with primary refractory disease may be achieved in approximately one-half with second line chemotherapy that incorporates drugs not used in initial treatment followed by high dose chemotherapy and autologous hematopoietic cell rescue (HDT and SCT). The treatment of relapsed disease primarily consists of combination chemotherapy with or without radiotherapy; radiotherapy alone is usually not used. Salvage therapy with second or third line regimens can achieve responses in approximately 50% of these patients, although long-term disease-free survival after the treatment of relapse with chemotherapy alone is less common. The choice of therapy is usually based upon prognostic features and patients with a localized, asymptomatic relapse occurring more than 12 months after the initial treatment are usually treated with conventional salvage chemotherapy, often combined with radiation therapy with or without HDT and autologous SCT. The value of HDT is uncertain in this group and may be unnecessarily toxic. High dose chemotherapy and autologous SCT should be considered as the treatment of choice for treatment of early relapses (less than 12 months after treatment) or induction failures, second relapses after conventional treatment for first relapse or generalized systemic relapses even beyond 12 months.^7^–^12^ However, it is of great importance that complete remission is achieved prior to transplantation. This is supported by emerging experience with PET scans obtained at the end of salvage chemotherapy, but prior to high-dose therapy with autologous transplant. In this setting, a negative scan has a markedly positive predictive value with 93% progression-free survival at two years in one series.^13^ In comparison, the majority of PET-positive patients relapse despite high-dose therapy.

Radiation therapy is in the relapsed setting indicated for patients with localized residual disease after salvage chemotherapy. In addition, patients with a localized late relapse may achieve long-term remission with chemotherapy followed by involved-field radiation therapy (IF-RT) rather than chemotherapy followed by HDT. The role of IF-RT in the treatment of patients who achieve a complete response to chemotherapy and plan to proceed with HDT is less clear.^6^,^14^,^15^

Risk factors for relapse after second line therapy include not only the patient and tumor specific markers (*e.g.*, CD68 expression), but also the initial response to therapy and the duration of this initial response. The German Hodgkin’s Lymphoma Study Group (GHSG) identified three adverse risk factors for second relapse following various forms of salvage therapy, which included hematopoietic cell transplantation in one-third of the cases. These adverse risk factors included time to first recurrence of less or equal to 12 months, stage III or IV disease at first relapse and anemia at the time of first relapse.^1^

Patients who relapse following autologous SCT have limited treatment options. A second autologous SCT is an option only in highly selected patients because of their limited bone marrow reserve and due to the fact that the disease that relapses after a first autologous SCT is generally more chemotherapy resistant.^16^ Single agent chemotherapy is often used in this setting, but there are no guidelines for the selection of agents. Other options include the use of brentuximab vedotin, bendamustine, rituximab, mTOR inhibitors (*e.g.*, everolimus), immunomodulators (*e.g.*, lenalidomide), histone deacetylase inhibitors, adoptive immunotherapy, local regional irradiation, or allogeneic SCT.^17^–^26^ In addition, the anti PD-1 antibody nivolumab has shown quite impressive responses in patients with HL who relapsed after prior autologous SCT.^27^ Allogeneic SCT is usually offered to patients with HL as a salvage therapy following relapse or progression after autologous SCT. Long term remissions can be obtained after allogeneic SCT in a select group of patients. An advantage of an allogeneic SCT graft is the use of tumor-free graft and the transfer of robust immune system from a healthy donor that can mediate the “graft versus lymphoma” effect.

## Brentuximab vedotin

**FIGURE 1.**
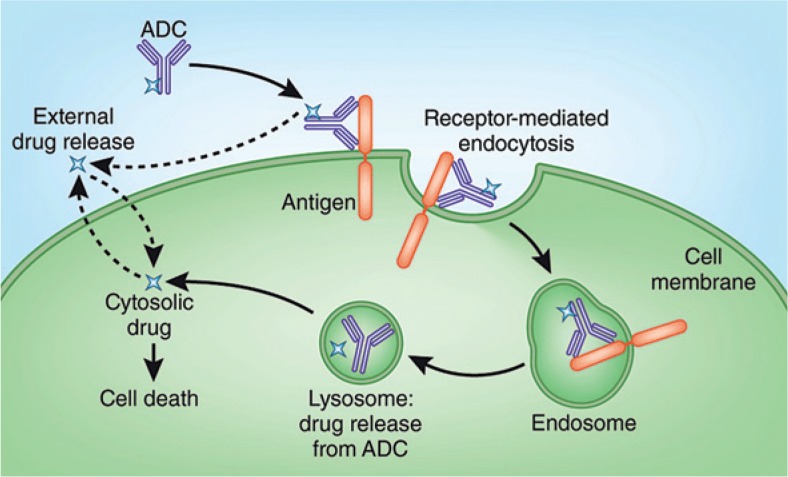
Mechanism of action of brentuximab vedotin. ADC = antibody-drug conjugate

Brentuximab vedotin (SGN-35) is an immunotoxin composed of a CD30 directed antibody linked to the antitubulin agent monomethyl auristatin E (MMAE).^17^,^28^ The antibody drug conjugate enables the antibody to selectively deliver a chemotherapy agent to CD30 positive tumor cells. The antibody and MMAE are joined together by a protease-cleavable linker that is stable in plasma but degraded by lysosomal enzymes. This conjugate binds to CD30, which is expressed on the surface of HL cells, then gets internalized and traffics to lysosome, where the MMAE is released. The MMAE then disrupts the microtubule network, leading to cell cycle arrest in G2/M phase and apoptosis (Figure 1).

Brentuximab vedotin has been approved by FDA and later EMA for the treatment of patients with relapsed or refractory CD30+ HL after failure of autologous hematopoietic SCT or after failure of at least two prior therapies in patients who are not candidates for HDT and autologous SCT or multi-agent chemotherapy.^29^ A subset of patients treated with brentuximab vedotin in this setting has been able to proceed with successful reduced intensity conditioning followed by allogeneic SCT.^30^ This drug is effective and has been approved also in relapsed or refractory systemic anaplastic large cell lymphoma (sALCL).

Several studies have demonstrated the activity of brentuximab vedotin in patients with HL.^17^,^31^–^36^ In a phase I trial SG035-0001 of brentuximab vedotin in 45 patients with CD30 positive hematopoietic cancers (42 of whom had HL) relapsed or refractory after a median of three prior therapies, there were 11 complete remissions (CR), six partial remissions (PR), and 19 cases with stable disease.^31^ Overall response rate in patients receiving 1.8 mg/kg was 50% and median duration of objective response was 9.7 months with a median progression-free survival of 5.9 months. Side effects were generally mild with the most common being fatigue, pyrexia, diarrhea, nausea, neutropenia, and peripheral neuropathy. The dose limiting toxicity at 2.7 mg/kg was acute renal failure, hyperglycemia, prostatitis, and febrile neutropenia. The maximum tolerated dose was defined as 1.8 mg/kg i.v. every three weeks.

Another phase I trial SG035-0002 was a dose-escalation study (0.4–1.4 mg/kg) of brentuximab vedotin given on days 1, 8, and 15 of 28-day cycles.^37^ Forty-four patients with relapsed or refractory CD30+ hematological malignancies were included, 38 of them with relapsed or refractory HL, 68% of all patients were previously treated with HDT. The maximum tolerated dose was defined as 1.2 mg/kg, the most common side effects were peripheral sensory neuropathy (66%), fatigue (52%), and nausea (50%). The overall response rate for all dose groups was 59% (34% CR), and with a median follow-up of 45.1 weeks the median progression-free survival was 28.7 weeks and the median overall survival has not been reached yet.

In a pivotal phase II multicenter trial SG035-0003, 102 patients with relapsed or refractory HL after prior autologous SCT were treated with brentuximab vedotin (1.8 mg/kg every three weeks for up to 16 cycles).^17^ The overall response rate was 75% (34% CR) with median times to objective response and CR of 5.7 and 12 weeks, respectively. Median durations of response were 20.5 and 6.7 months for those with CR and all responding patients, respectively. At a median follow-up of 18.5 months, the median progression-free survivals were 21.7 and 5.6 months for patients with CR and all patients, respectively. After responding to brentuximab vedotin, eight patients received an allo-SCT as their first subsequent therapy - five patients with CR and three patients with PR. All eight patients were alive and remained in follow-up at the time of data cutoff. The most common non-hematologic toxicities were peripheral sensory neuropathy (42%), nausea (35%), and fatigue (34%). The most common severe (grade 3/4) side effects were neutropenia (20%), thrombocytopenia (8%), anemia (6%), and peripheral sensory neuropathy (8%).

The data of the SG035-0003 were updated with a median follow-up of 33.3 months post first brentuximab vedotin dose.^38^ The updated median overall survival and progression-free survival were estimated at 40.5 months and 9.3 months, respectively. Improved outcomes were observed in patients who achieved a CR, with estimated 3-year overall survival and progression-free survival rates of 73% and 58%, respectively, in this group (and medians not reached). Of the 34 patients who obtained CR, 16 (47%) remained progression-free after a median of 53.3 months of observation, 12 patients remained progression-free without a consolidative allogeneic stem cell transplant. Those patients in remission tended to be younger, predominately females, diagnosed with HL for a shorter period prior to receiving brentuximab vedotin, having a relapsed rather than refractory disease, with a lower ECOG performance score and a smaller disease burden prior to enrollment. They also received more cycles of brentuximab vedotin but with an equal incidence and severity of adverse events relative to other patients.

In another phase II multicenter trial, 25 patients with relapsed HL after prior allogeneic SCT were treated with brentuximab vedotin (1.2 mg/kg or 1.8 mg/kg every three weeks for up to 16 cycles).^32^ The overall response rate was 50% (38% CR) with a median time to response of 8.1 weeks. At a median follow-up of 34 weeks, the median progression-free survival was 7.8 months and the median overall survival has not been reached. The most common toxicities were cough, fatigue, and pyrexia (52% each), nausea and peripheral sensory neuropathy (48% each), and dyspnea (40%). The most common severe (grade 3/4) toxicities were neutropenia (24%) and anemia (20%).

Rothe *et al.*^39^ reported effectiveness and safety in 16 patients with HL (14 patients) or sALCL (2 patients) who have not undergone a prior HDT from GHSG enrolled in a named patient program with brentuximab vedotin 1.8 mg/kg every three weeks. Five patients achieved CR and 6 patients PR giving an overall response rate of 69%. Six of the 16 patients proceeded to HDT with autologous transplant in 4 and allogeneic transplant in 2 patients. The 12-month overall survival for all patients was 68% and the 12-month progression-free survival 22%. The toxicity profile was similar as reported in previous studies.

Rothe *et al.*^33^ also evaluated brentuximab vedotin in relapsed and refractory HL. Results were pooled from the multicentric study by the GHSG including 45 HL patients (34 patients from the named patient program) and SGN35-007 study (11 patients), 87% of them receiving a previous transplant. The overall response rate was 60%, with 22% of CR and 38% of PR. The median progression-free survival was 8 months and the progression-free survival at 12 months 43% with an overall survival of 83% at 12 months and median overall survival not reached. Grade 3–4 toxicities were of similar frequencies as reported before - there were 13% of neutropenia/sepsis, 7% of thrombocytopenia, and 7% of infections with no grade 3–4 neuropathy reported.

Brentuximab vedotin is usually well tolerated with an acceptable toxicity profile in these usually heavily pretreated patients.^17^,^31^–^33^,^37^–^43^ Infusion reactions are uncommon but severe anaphylaxis has been reported.^29^ Peripheral polyneuropathy is one of the most common side effects of brentuximab vedotin treatment with 53% of patients experiencing at least one treatment emerging event. However, in 80% of these patients the neuropathy presented as partially or completely reversible.^17^ Concomitant administration of brentuximab vedotin and bleomycin is contraindicated due to unacceptable pulmonary toxicity. Progressive multifocal leukoencephalopathy (PML) and acute pancreatitis are rare but potentially fatal complications of brentuximab vedotin treatment.^42^,^43^ In PML, other possible contributory factors beside brentuximab include prior therapies and underlying disease that may cause immunosuppression.^41^

Brentuximab vedotin has been available since 2012 also in Slovenia. Up till now, we have treated five adult patients with relapsed or refractory HL. All of them were heavily pretreated-in three patients brentuximab vedotin was more than third line treatment (in two of them being the seventh line of treatment), in one patient it was given following the autologous and in one patient following the allogeneic SCT. Patients received from 3 to 16 cycles of brentuximab but no CRs were achieved-there were two PRs and in two patients the disease progressed while the fifth patient is still under treatment awaiting first treatment evaluation. The treatment was very well tolerated in all patients and no dose reductions were needed except in the fifth patient who is receiving brentuximab vedotin at a reduced dosage due to a preexisting neuropathy. Regarding the inferior effectiveness in our patients in comparison with the one reported in the above studies^17^,^31^,^38^ we conclude that it must be on account of the heavy pretreatment of all our patients suggesting an earlier use of brentuximab vedotin.

## Conclusions

Patients with relapsed of refractory HL have limited treatment options - especially those who relapse after HDT and autologous SCT or those who have failed at least two prior multi-agent chemotherapy regimens and are not candidates for HDT. Brentuximab vedotin represents an additional treatment option with substantial effectiveness and acceptable toxicity in this select population.
